# Supplementary material for: Regional sequence expansion or collapse in heterozygous genome assemblies
Source: PLoS Comput Biol. 2020 Jul 31;16(7):e1008104. doi: 10.1371/journal.pcbi.1008104 (PMC7423139; doi:10.1371/journal.pcbi.1008104)
Supplement: Attachment — Submitted filename: Response to Reviews of PLOS Computational Biology.pdf [file pcbi.1008104.s006.pdf]

Dear Dr. Ouzounis and Dr. Noble,

We appreciate the careful attention to detail and thorough reviews. We have made edits as discussed below in blue font. We have repeated essentially all the analyses in light of reviewer comments, particularly to re-evaluate our choice of sequence length cutoff. In the process of re-annotating the genomes with a 200 bp size cutoff we observed unexpected findings which increase the value of our work in the larger genomics community and substantially improve the manuscript. We discuss below.

Dear Dr Bracht,

Thank you very much for submitting your manuscript 'Regional sequence expansion or collapse in heterozygous genome assemblies' for review by PLOS Computational Biology. Your manuscript has been fully evaluated by the PLOS Computational Biology editorial team and in this case also by independent peer reviewers. The reviewers appreciated the attention to an important problem, but raised some substantial concerns about the manuscript as it currently stands. While your manuscript cannot be accepted in its present form, we are willing to consider a revised version in which the issues raised by the reviewers have been adequately addressed. We cannot, of course, promise publication at that time.

Please note while forming your response, if your article is accepted, you may have the opportunity to make the peer review history publicly available. The record will include editor decision letters (with reviews) and your responses to reviewer comments. If eligible, we will contact you to opt in or out.

Your revisions should address the specific points made by each reviewer. Please return the revised version within the next 60 days. If you anticipate any delay in its return, we ask that you let us know the expected resubmission date by email at ploscompbiol@plos.org. Revised manuscripts received beyond 60 days may require evaluation and peer review similar to that applied to newly submitted manuscripts.

In addition, when you are ready to resubmit, please be prepared to provide the following:

(1) A detailed list of your responses to the review comments and the changes you have made in the manuscript. We require a file of this nature before your manuscript is passed back to the editors.

(2) A copy of your manuscript with the changes highlighted (encouraged). We encourage authors, if possible to show clearly where changes have been made to their manuscript e.g. by highlighting text.

(3) A striking still image to accompany your article (optional). If the image is judged to be suitable by the editors, it may be featured on our website and might be chosen as the issue image for that month. These square, high-quality images should be accompanied by a short caption. Please note as well that there should be no copyright restrictions on the use of the image, so that it can be published under the Open-Access license and be subject only to appropriate attribution.

Before you resubmit your manuscript, please consult our Submission Checklist to ensure your manuscript is formatted correctly for PLOS Computational Biology: http://www.ploscompbiol.org/static/checklist.action. Some key points to remember are:

- Figures uploaded separately as TIFF or EPS files (if you wish, your figures may remain in your main manuscript file in addition).

- Supporting Information uploaded as separate files, titled Dataset, Figure, Table, Text, Protocol, Audio, or Video.

- Funding information in the 'Financial Disclosure' box in the online system.

While revising your submission, please upload your figure files to the Preflight Analysis and Conversion Engine (PACE) digital diagnostic tool, https://pacev2.apexcovantage.com PACE helps ensure that figures meet PLOS requirements. To use PACE, you must first register as a user. Then, login and navigate to the UPLOAD tab, where you will find detailed instructions on how to use the tool. If you encounter any issues or have any questions when using PACE, please email us at figures@plos.org.

To enhance the reproducibility of your results, we recommend that you deposit your laboratory protocols in protocols.io, where a protocol can be assigned its own identifier (DOI) such that it can be cited independently in the future. For instructions see here.

We are sorry that we cannot be more positive about your manuscript at this stage, but if you have any concerns or questions, please do not hesitate to contact us.

Sincerely,

Christos A. Ouzounis

Associate Editor

PLOS Computational Biology

William Noble

Deputy Editor

PLOS Computational Biology

A link appears below if there are any accompanying review attachments. If you believe any reviews to be missing, please contact ploscompbiol@plos.org immediately:

Reviewer's Responses to Questions

**Comments to the Authors:**

**Please note here if the review is uploaded as an attachment.**

Reviewer #1: OVERVIEW

The development of efficient and inexpensive next-generation genome sequencing has enabled an explosion of new genome sequences for 'non-model' organisms. Such organisms are either not studied much in laboratories as a matter of past custom, or practically not feasible for such study, but their genomes are of biological importance nevertheless. Of their nature, such

organisms are often outbred with substantial genomic diversity. Even for small sample populations used to extract the genomic DNA used for sequencing and assembly; levels of genetic heterogeneity can reach the 'hyperdiverse' level (e.g., 7% variation in non-selected nucleotides of the nematode Caenorhabditis brenneri) and almost always contain substantial amounts of unresolved allelism.

Asalone et al. have recently characterized the genome for a nonmodel but biologically interesting subterranean nematode, Halicephalobus mephisto. In so doing, they have identified a potential artifact of genomic analysis that to my knowledge has not been previously described: depending on fine details of the genome assembly programs and parameters used, different regions of the genome encoding gene families with biologically interesting functions can assemble in two different ways. They can either assemble so that two or more alleles are compressed in silico into a single sequence, or instead be assembled so that two or more alleles are artifactually linked in a tandem sequence array. Given heterozygosity throughout a genome, such variable compression or tandem expansion can have a visible effect on what genes are predicted for a genome, with expansions or compressions of a gene family having downstream effects on its biological function being scored as over- or under-represented among the protein-coding genes of that genome.

The authors compare assemblies from Platanus versus SOAPdenovo2 versus their best reference assembly (generated by Platanus with PacBio long-read superscaffolding). They observe a general tendency for genome assembly regions with lower polymorphism (assayed by raw Illumina reads mapped to a given assembly) to correlate with greater length. They observe striking differences between assemblies in which heterozygous regions are represented, despite those assemblies having considerably more similar total lengths. The authors conclude that, in assessing quality of a genome assembly, it is not sufficient to look at the size-weighted median of its scaffold or contig sizes (i.e., its N50 score); it is also advisable to assess its degree of sequence coverage and heterozygosity, with caution being exercised for regions of the assembly showing abnormally high or abnormally low heterozygosity (and, in parallel, abnormally low or abnormally high coverage).

One general result: given heterozygous sequence data, Platanus seriously outperforms SOAPdenovo2. The numbers in Supplemental Table 1 make that quite clear. Although the authors do not provide results for other mainstream short-read assemblers comparable to SOAPdenovo2 (e.g., ABySS 2), their results make it advisable that researchers assembling short reads from a heterozygous organism use a heterozygosity-aware program such as Platanus.

Although the general points are the paper is well-taken, I have some specific questions and caveats about it, along with some suggested revisions.

SPECIFIC QUESTIONS AND CAVEATS

An inconspicuous-looking point of the Methods may be driving nontrivial amounts of the differences between how different genome assemblies are scored for completeness: the authors have imposed a minimum scaffold/contig size of 1,000 nt for all of their competing assemblies. This is likely to be harmless for those assemblies with high N50 values, but may be leading to substantial losses of sequence information for those four assemblies with low N50 values (Platanus step-size 1, N50 = 2.8 kb; SOAPdenovo2 k-mer 23, N50 = 2.7 kb; SOAPdenovo2 k-mer 47, N50 = 1.9 kb; and SOAPdenovo2 k-mer 63, N50 = 1.9 kb) -- particularly when one considers that the N50 values given in Fig. 1 and Supp. Tab. 1 were computed for these assemblies *after* scaffolds/contigs of <1 kb had been discarded. If the authors had performed their analyses on assemblies that had had a less stringent minimum size filter (such as 200 nt), how much would the downstream results change? This question clearly has to affect BUSCO scores (Figure 1), but could conceivably also affect evidence-based annotation of genes (Figure 3) and homology of genes to other genes (Figure 4), since assemblies with low N50 values are likely to have fragmented or partial gene predictions.

The reviewer makes an insightful point here. We have exhaustively re-performed our analyses with a 200 bp size cutoff and included these data in the revised manuscript. Now, the differences between the 1000 bp and the 200 bp size cutoff data indicate which sequences are highly fragmented. One striking finding from this was that when the 200 -1000 bp sequences are included, there is a huge ballooning of protein predictions from Maker2 in the fragmented assemblies. The increase is far more than the 2-fold we would have predicted from allelic separation but points to a new phenomena, potentially the combinatorial “spreading” of possible alleles. If there are snps in close proximity, the assembler is potentially constructing every possible phased path between snps in the de Bruijn graph. This is a phenomena that is novel and important to document. We also found that the pgp protein family (Figure 6, now Figure 5) was more complete with the 200 -1000 bp sequences included, and the percent grouping by OrthoMCL is lower for the fragmented assemblies is lower as they contain many more short, non-group-able sequences. The BUSCO results change only slightly because many of the 200-1000 bp sequences are too short to annotate well.

At crucial points in their Methods -- specifically, when they compute heterozygosity levels for an entire genome assembly, or for particular genes within that assembly -- the authors invoke

nameless "custom python scripts". Given the central importance of this computation to their work, this is entirely unacceptable. Each Python script used in the work must be given a name in the Methods and must be explicitly available through either github or some equivalently useful public software repository. Note: I am aware that the authors have written "All python scripts are available from the github database (repository: "Name TBD").", but that is not enough!

We appreciate the reviewer's comment: open access is critically important and our custom pipelines may help other workers. We have uploaded all scripts into https://github.com/brachtlab/Regional_heterozygosity where they are publicaly available. The names of the Python scripts have also been added to the text.

The authors cite results based on 11 alternative (non-reference) genome assemblies for H. mephisto. It would be preferable if these genome assemblies were themselves publicly available in some data repository. One data repository that works quite well for permanent archiving of such data is the Open Science Foundation (https://osf.io). Other options are Figshare (https://figshare.com) and Zenodo (https://zenodo.org).

We appreciate the comment and have published all assemblies on Zenodo at https://zenodo.org/record/3738267#.XoYGU9NKj_8. Note, since we used both 1 kb size cutoff and a 200 bp size cutoff per reviewer comments, we have uploaded both versions of each assembly (22 total assemblies).

The authors have devised their own tools for making either genome-wide or regional estimates of nucleotide heterozygosity. This is ingenious and potentially valuable to other researchers. However, there already exists a published open-source programs for estimating overall heterozygosity of a given organism, directly from that organism's raw Illumina sequence read set: GenomeScope (https://github.com/schatzlab/genomescope.git and https://academic.oup.com/bioinformatics/article/33/14/2202/3089939). I think it would be highly desirable for the authors to compute heterozygosities for H. mephisto from their raw Illumina sequence reads using either GenomeScope or an equivalent k-mer analysis tool, and then for them to compare the heterozygosity score generated with one of these tools versus their own results.

We have run GenomeScope on our raw data and include that result in our revised manuscript. It agreed with the numbers we were getting from our read-mapping approach.

SUGGESTED REVISIONS

The authors had no page numbers in their manuscript. Next time, please have them! Page numbers in manuscripts help readers (even though the readers in this case will be a small

number of editors and reviewers.) In this case, for clarity while reviewing, I am providing page numbers using my own count (with the title and abstract being on page 1).

We have added page numbers.

Page 4 --

"(Borgonie et al., 2011)": although cited in the text, this was not included in the References on pp. 18-22. I assume that the authors meant Borgonie et al. (2011), Nature 474, 79-82, PubMed 21637257. Please add this reference to the References; more importantly, please proofread the entire manuscript to ensure that there are no other missing references cited anywhere.

Citation added.

Page 5 --

Legend for Figure 1: "N50, heterozygosity, and the BUSCO results." I would prefer something like "N50 (in nt), heterozygosity (as defined in Methods), and the BUSCO results." As it stands, the reader is left to guess what the measurement unit for N50 is, and to wonder where the heterozygosity comes from. It will be good for readers to understand that the authors are using their own methods of computing heterozygosity rather than using previously published methods.

Done.

Page 6 --

"We found that N50 is highly correlated with evidence-supported genes predicted..." What are the mean and median sizes of protein-coding sequences for these genes, and how do they vary with respect to assembly N50? It is a long-known problem in genome analysis that assemblies with low N50 values result in gene predictions that are fragmentary or partial; fragmentary or partial gene predictions, in turn, may lower the rate at which genes are scored as evidence-supported. (The same caveat also applies to Figures 3 and 4, which are cited at this point in the text.)

The point is a good one. We have now included two new tables for protein predictions that include the differences in lengths for evidence-supported and non-supported. In sum, we found

statistically robust differences with the non-evidence proteins being shorter (and many more of them in the 200 bp size cutoff assemblies as you might expect).

"However, we found that..." To avoid awkwardly starting two sentences in a row with "However", I suggest that this instance of "However" be replaced with something like "Nevertheless".

Done.

Page 7 --

"we extracted the second-largest group of proteins": why was the *second*-largest group chosen? Why not the first, or the third? The answer could go here or in Methods.

We chose the second, now fifth, because the first grouped family was too large to build an interpretable tree. We have included that information in the revised manuscript. As it is, the tree has 167 sequences and requires a zoom-in box to visualize one clade.

Page 10 --

The authors write: "We would predict that if an assembler maximally 'spreads out' the variation within a dataset into distinct contigs, coverage and length assembled would go up, while heterozygosity would go down as the reads are able to find their perfect match."

Unless I have misunderstood the argument of this paper badly, this is not quite correct, and they should have instead written: "We would predict that if an assembler maximally 'spreads out' the variation within a dataset into distinct contigs, length assembled would go up, while coverage and heterozygosity would go down as the reads are able to find their perfect match."

The reviewer is exactly correct. We thank the reviewer for catching this typo. We have corrected the manuscript.

Page 11 --

"smaller than 1kb" should be "smaller than 1 kb" (i.e., do not fuse a number and its measurement unit).

Done.

Page 12 --

"These assembly variations are not easily detected particularly when assembling a genome for the first time" should read "These assembly variations are not easily detected, particularly when assembling a genome for the first time".

Done.

Pages 12 and 13 --

"sequences lower than 1000bp were removed prior to subsequent analysis", and "Sequences smaller than 1000bp were removed from these assemblies prior to downstream analysis". First, replace '1000bp' with '1000 bp'. Second, this filtering step can have strong and differential effects on genome assembly analysis. Consider the assembly N50s listed in both Figure 1 and Supplemental Table 1. For the reference genome (N50 = 313 kb), the effect of discarding scaffolds or contigs of under 1 kb will be slight -- almost all of the assembly will be over that threshold anyway. However, for four of the most fragmented genome assemblies (Platanus step-size 1, N50 = 2.8 kb; SOAPdenovo2 k-mer 23, N50 = 2.7 kb; SOAPdenovo2 k-mer 47, N50 = 1.9 kb; and SOAPdenovo2 k-mer 63, N50 = 1.9 kb), filtering out sequences of <1 kb is likely to be substantially depleting genomic contents -- particularly since these low N50s were presumably computed *after* sequences of <1 kb had been filtered out.

We agree with the reviewer and as mentioned, we have re-done the analysis with a 200 bp size selection and updated Figure 1 and supplemental tables.

Given that the authors observe profound drops in their %BUSCO scores for these very same four assemblies (Figure 1 and Supp. Table 1), it is difficult not to suspect that they might have observed significantly better %BUSCO scores if they had adopted a somewhat smaller minimum scaffold/contig size (say, 200 nt instead of 1,000 nt). That, in turn, raises the question

of how many *other* results in this paper would be significantly changed if the minimum size had been so lowered.

We have changed the cut off to 200 nt but the BUSCO scores did not significantly change, as updated in the new Figure 1. The number of complete by BUSCO only varied by an average of 2.5% across all assemblies and around 3% for the four assemblies in question. We note throughout the manuscript that some other analyses did significantly change so we appreciate the importance of this size cutoff.

Page 14 --

"H. Mephisto" should read "H. mephisto".

Done.

Page 15 --

"SamTools" should probably be written "Samtools" (following how it is written on the author's main software page -- see http://www.htslib.org).

Done.

"BCFTools" should be written "BCFtools" (again, following http://www.htslib.org).

Done.

"variants were called using the mpileup and call function" should read 'functions', not 'function'; also, from exactly which software suite were these functions taken? The way the sentence is written, it is not clear whether they are from SAMtools or BCFtools.

Done.

"10kb" should be "10 kb".

Done.

Page 16 --

"(Note that the dynamics..." starts with a parenthesis ['('], but does not close with one [')'].

Removed the [(].

Figure 1 --

Please revise the header "N50" to "N50 (nt)", so that the reader knows what size the N50s are in.

Done.

Please *add* a column for total genome assembly sizes (i.e., total genome assembly lengths). I know that these data are in Supplemental Table 1, but I think they would be significantly useful in Figure 1, which is what most readers will see. The genome assemblies should be rounded to 0.1 Mb, and the header should be something like "Genome size (Mb)".

Done.

Figures 3 and 4 --

For genes predicted in the various H. mephisto assemblies, these two figures show quite different rates of evidence-association (as scored by MAKER; Figure 3) and homology to other genes (as scored by OrthoMCL; Figure 4). The authors note that different assemblies can have similar numbers of predicted genes, but quite different values for evidence-association or homology. However, they do not show whether these genes vary in the mean or median length of their protein-coding sequences; yet it is quite likely that the four genome assemblies with lowest N50 values (under 3.0 kb) will have significant numbers of truncated or partial gene predictions, which may well affect both assays. I would like to see the authors address this point in some reasonable way.

We have analyzed the length differences in evidence-supported vs. non-supported predictions. As might be expected the non-supported consistently have lower mean and median lengths and there is strong statistical support for the difference between the two groups. This information is in the revised manuscript.

Figure 4 --

This figure shows different assemblies as "Platanus", "Platanus and PacBio", or "SOAPdenovo2". However, I would prefer to have individual labels next to each glyph, specifying exactly which assembly is associated with each data point in the figure (for instance, *which* Platanus assembly gave rise to the unpromising data point with only ~7,250 predicted proteins and ~0.93 proportion grouped?).

The original Figure 4 has been removed because we realized its data is covered by the subsequent figure. However we have incorporated the reviewer's comment in a revised Figure 5 (now a new Figure 4) showing the individual assemblies as clearly labeled points.

Also, the x-axis lists "proteins". However, not all gene prediction methods give exactly one predicted protein isoform per gene; my guess is that there is such a relationship, in this instance, but my guess could be wrong. The authors should make it clear in the legend for this figure that there is (or, is not) a one-to-one relationship between proteins in this figure and genes predicted in the various assemblies.

We originally ran Maker2 with alternative splicing turned off, but this comment motivated a manual examination of the data just to be sure, since apparently the algorithms within Maker can still identify isoforms even if the Maker2 setting is off. We confirmed that there were not isoforms predicted in our Maker outputs, with one sole, minor exception. This exception consists of 3 cases of alternative isoforms that were predicted by exonerate in the SOAPdenovo2 k-mer 63 assembly (which yielded 54,877 total predictions). In that case there were two cases of 2-isoform genes and a case of one 3-isoform gene. Since the 200 bp assemblies yielded 330,292 total protein predictions, the isoform-driven expansion is 0.001% of the total. The effect is completely negligible for our purposes and does not alter our conclusions. (None of the multi-isoform genes were pgp proteins so do not account for duplications in the tree shown in Figure 5).

Supplemental Table 2 --

Here, it would be good to add a column for the value "Observed/Expected" (i.e., the ratio of the existing "Observed Hits" and "Expected Hits" columns.) Adding such a column would allow readers to sort the Excel spreadsheet by this ratio, and thus get a clear view of which particular PANTHER functions are either most overrepresented or most underrepresented by the various genome assemblies. (They can already use the 'sort' function in Excel to reorder the PANTHER functions by ascending "Raw P-value" scores, and thus get a clear view of which over- or under-represented functions are most statistically significant.)

Done.

Reviewer #2: In this manuscript, Asalone et al. examine the effects of assembler choice and parameter values on genome assembly of diploid genomes with high levels of heterozygosity. Specifically, they examine assemblies generated for a nematode species, Halicephalobus mephisto, using two different assemblers (Platanus and SOAPdenovo2) with various parameter settings. Assemblies are compared with a reference assembly generated using additional PacBio data and the Platanus assembler. Assemblies are evaluated with BUSCO, alignments with the reference genome, numbers of predicted protein-coding genes, and enrichment/depletion analysis of protein function groups with respect to the reference genome protein set. The overall conclusion of this work is that assemblies can vary significantly in erroneously expanded or contracted regions even if other measures of assembly quality are consistently good.

The topic of assembly accuracy in the presence of high heterozygosity is an important one and thus this is a welcome contribution. Whereas the overall conclusions of the paper are supported by the experiments, I found the experiments and methods to be confusing and perhaps overly complicated.

Specific comments:

1. Nowhere in the manuscript is a description of the underlying data that was assembled. After some digging through the references, I'm assuming it was the Illumina data described in Weinstein et al. 2019, but this needs to be clear and explicit in this paper. There is also mention

of "RNA from H. mephisto", by which I'm assuming the authors mean RNA-seq data, but there is no description of these data anywhere.

The reviewer is correct. We have added information to clarify this in the manuscript. The Illumina and RNAseq data are publicly available on the Sequence Reads archive at PRJNA528747 and the details are given in a new Data Availability section we have added to the manuscript.

2. It seems troubling that one of the assemblers evaluated was the same one used to generate the "reference" assembly. And as I understand it, PacBio reads were only used for scaffolding this reference assembly, and not for constructing the original contigs, and thus erroneous expansions or contractions made by Platanus on the Illumina data are not necessarily corrected by the PacBio data in this reference assembly. This issue needs clarification and discussion in this manuscript. In particular, an assembly that appears to have an enrichment or depletion of a certain protein functional group relative to the reference is not necessarily less accurate, because the reference may (perhaps equally likely) be in error with respect to this group.

We have added a thorough discussion of this issue to the text.

3. The evaluation of expansion/contraction via enrichment/depletion of functional groups seems more indirect and complicated than necessary. Why not simply align the genomes (gene sets) pairwise to the reference and quantify how many genes/regions are expanded/contracted with respect to the reference? One would expect only expansion/contraction of highly-similar sequences, not of broad functional categories of proteins.

While the reviewer's suggestion seems reasonable on its surface, we suspect they may not have appreciated the challenges involved in implementation. For example blastn only records matches (above a statistical threshold), and not what is missing.. Furthermore, the complexity quickly multiplies when faced with 1) paralogy within the genome, 2) varying levels of fragmentation across multiple alternate assemblies, each of which has 3) varying levels of expansion or contraction due to heterozygosity. Distinguishing excess fragmentation versus expansion of heterozygosity as an explanation for an apparent duplication would be quite challenging. As part of our future directions we envision an 'assembly scanner' which could look across an assembly and, using the raw sequencing data, score regions as expanded, contracted, or confidently normal. However, implementing such a tool is beyond the scope of the current manuscript.

All this being said, our analysis of LAST and overall missing sequences is one approach that begins to address the reviewer's suggestion, and that is already part of the current manuscript, but as outlined above, a more detailed method is part of the next project.

4. There is no logic given for why the an assembly with a high (or low?) proportion of grouped proteins by OrthoMCL would be better/worse than another assembly.

We have added an explanation that notes non-grouped proteins are unique and in our case are often short. This suggests that low proportion of grouped proteins shows more fragmented assemblies.

5. Please provide a definition for an "evidence-supported gene"

Definition has been added.

6. I have never heard of an isolog or iso-ortholog. Perhaps simply one-to-one ortholog can be used instead.

We invented the term and it refers to paralogs that are identical because they are the *exact same gene* recovered from different assemblies. They do not represent genomic copies, but rather are literally the *same gene*. Hence the iso- prefix. We have added text to the manuscript to clarify this.

7. Please describe early on how heterozygosity is defined/measured in these genome assemblies.

A brief description of how heterozygosity is measured was added to the methods.

8. Fig 2 - the dot plots not very informative. They would be greatly improved if assembly contigs were ordered and oriented according to the reference.

We thank the reviewer for the suggestion and have adjusted the figure accordingly.

9. Fig 5 - there are so few points here - just show the points instead of a box plot.

We have adjusted this to a jitter plot and is now figure 4.

10. The Borgonie et al. 2011 reference seems to be missing.

Done.

11. Benchmarking *University* Single Copy Orthologs => universal

Done.

12. The GitHub link to the software/scripts used is not provided.

The Github repository is available at: https://github.com/brachtlab/Regional_heterozygosity and has been included under Data Availability in the manuscript.

---

**Have all data underlying the figures and results presented in the manuscript been provided?**

Large-scale datasets should be made available via a public repository as described in the *PLOS Computational Biology* data availability policy, and numerical data that underlies graphs or summary statistics should be provided in spreadsheet form as supporting information.

Reviewer #1: Yes

Reviewer #2: Yes

---

PLOS authors have the option to publish the peer review history of their article (what does this mean?). If published, this will include your full peer review and any attached files.

If you choose “no”, your identity will remain anonymous but your review may still be made public.

**Do you want your identity to be public for this peer review?** For information about this choice, including consent withdrawal, please see our Privacy Policy.

Reviewer #1: No

Reviewer #2: No
