# Supplementary material for: Regional sequence expansion or collapse in heterozygous genome assemblies
Source: PLoS Comput Biol. 2020 Jul 31;16(7):e1008104. doi: 10.1371/journal.pcbi.1008104 (PMC7423139; doi:10.1371/journal.pcbi.1008104)
Supplement: Attachment — Submitted filename: Response to Reviews v3 (1).pdf [file pcbi.1008104.s007.pdf]

Dear Dr. Ouzounis and Dr. Noble,

Thank you for your comments on our manuscript. We have responded and include comments in blue font below.

Sincerely,

John Bracht

Dear Dr. Bracht,

Thank you very much for submitting your manuscript "Regional sequence expansion or collapse in heterozygous genome assemblies" for consideration at PLOS Computational Biology. As with all papers reviewed by the journal, your manuscript was reviewed by members of the editorial board and by several independent reviewers. The reviewers appreciated the attention to an important topic. Based on the reviews, we are likely to accept this manuscript for publication, providing that you modify the manuscript according to the review recommendations.

Please prepare and submit your revised manuscript within 30 days. If you anticipate any delay, please let us know the expected resubmission date by replying to this email.

When you are ready to resubmit, please upload the following:

[1] A letter containing a detailed list of your responses to all review comments, and a description of the changes you have made in the manuscript. Please note while forming your response, if your article is accepted, you may have the opportunity to make the peer review history publicly available. The record will include editor decision letters (with reviews) and your responses to reviewer comments. If eligible, we will contact you to opt in or out

[2] Two versions of the revised manuscript: one with either highlights or tracked changes denoting where the text has been changed; the other a clean version (uploaded as the manuscript file).

Important additional instructions are given below your reviewer comments.

Thank you again for your submission to our journal. We hope that our editorial process has been constructive so far, and we welcome your feedback at any time. Please don't hesitate to contact us if you have any questions or comments.

Sincerely,

Christos A. Ouzounis

Associate Editor

PLOS Computational Biology

William Noble

Deputy Editor

PLOS Computational Biology

Reviewer's Responses to Questions

Comments to the Authors:

Please note here if the review is uploaded as an attachment.

Reviewer #1: I am fully satisfied with the response of the authors to my previous comments, and have no further questions or suggested revisions.

Reviewer #2: With this revision, the authors have satisfactorily addressed the majority of my previous comments. However, I continue to be of the opinion that a number of the analyses in this work are rather indirect and difficult to interpret.

1. In particular, the PANTHER analysis of enrichment/depletion of protein functional categories and the OrthoMCL grouping analysis are hard to interpret with regard to the quality of the assemblies. Consider one protein-coding gene in the reference genome and its assembly with one of the alternative assemblers or assembler parameters. There are many ways in which this gene might be assembled, but consider two simple erroneous cases: (1) the gene has two copies in the assembly and (2) the gene is fragmented into two non-overlapping pieces. In both cases, assuming protein-coding components can be detected in all contigs, there is an effective doubling of the gene, but only the former is truly an "expansion" of the gene in the assembly. It does not seem that either the PANTHER or OrthoMCL analyses can distinguish between these possibilities and thus the interpretation of their results is difficult. The OrthoMCL analysis is particularly hard to understand because an assembly that erroneously produced two copies of every gene would result in 100% grouping (because the two copies of each gene would fall into the same group), whereas an assembly that fragments each gene into many non-overlapping pieces that cannot be confidently aligned, would have a much lower % grouping. This seems to

be a roundabout way of assessing fragmentation but says little about expansion/contraction, which is the focus of the manuscript.

We appreciate this reviewer’s insightful comment, which inspired us to perform a proteome-wide analysis of fragmentation vs. duplication based on the OrthoMCL output. Because OrthoMCL’s output includes coordinates where matches to the reference assembly align, we were able to separate out fragmentation cases (non-overlapping alignments from a single assembly) vs. duplication cases (overlapping alignments from the same assembly). This is a bit complex because paralogy confuses the mapping and analysis, so we filtered paralogy out by removing cases where non-self reference matches occur. This led to a pretty clear picture of the relative ratio of fragmentation vs. duplication which we discuss in the revised text and which clearly shows that while fragmentation is detectable, and significant, it contributes less to the expansions than duplication across all assemblies. We feel this extra analysis is extremely valuable and we appreciate the reviewer’s comment which motivated us to investigate more thoroughly. We have made a new Figure 3C which includes this data. We have also clarified the OrthoMCL grouping section of the manuscript to make clear that sequence lengths is a driver of non-grouping as pointed out by the reviewer. Indeed (and in agreement with the reviewer), these grouping data highlight primarily the variance in protein sequence lengths and give no information on fragmentation or duplication per se.

2. The LAST analysis (alignment of each assembly to the reference) and associated Figure 2 is a much more direct and easier to interpret method of understanding expansion/contraction in an assembly compared to a reference. I recommend that the authors expand on this analysis. Briefly, LAST can used to identify the *single best place* in the reference to align each component of an assembly. I believe the authors are already using LAST for this purpose. Then, for each position in the reference genome, one can count how many positions in the assembly are aligned to it. The distribution of these counts is highly informative: the positions with zero alignments are "missing" (perhaps due to contraction) and positions with more than one alignment are duplicated/expanded in the assembly. This should be simple to implement and more directly assesses expansion/contraction/missing-ness than much of the rest of the analyses.

The Last algorithm has limitations for the number of sequences that can be aligned/plotted, and by plotting all the contigs from each assembly, the figure becomes too large and compressed to resolve the pattern of matching sequences. Indeed, while LAST users have discussed including an option to plot ‘missing’ sequences at the end of the Oxford grid, they have not implemented it owing to how much it would shrink the actual matching pattern, in exchange for very little meaningful information gain (lots of blank white space isn’t that meaningful). We have chosen to follow their lead, as we want to maximize the ability to see the matching patterns.

In addition, due to 200bp filtering for this analysis, as suggested by the reviewer, many of these contigs are short sequences with no alignments and therefore by plotting them we do not

believe it would show any additional information than the percent missing calculation which we provide in the figure. The percent missing is also given in Table S1 (for 200 bp size cutoff) and also for Table S2 (for 1000 bp size cutoff). We agree and understand the importance of quantifying these regions that are missing or have more than one alignment and have extensively documented this throughout the manuscript. In order to address this in Figure 2, we have added the percent missing and duplicated calculations directly to the panels of the figure.

3. Related to point 2 above, Figure 2 is quite important and could be improved. With a few tweaks, it can visually display expansions ("steeper" diagonals) and contractions/missing-ness ("less steep" diagonals). Suggested improvements are:

a. Clarify whether this is for the 200bp or 1000bp filtered assemblies. I would suggest using the 200bp assemblies so that one can still see if an assembly is relatively "complete" even if highly fragmented.

b. Keep the x-axis constant across all plots. It currently seems to be changing slightly between plots, which is misleading. All contigs in the reference should be plotted such that contigs that are missing in the assembly can be seen.

c. Include all contigs in the assembly on the y-axis, regardless of whether they have an alignment to the reference. That way one can visually see (1) how large the assembly is and (2) the fraction of the assembly that doesn't align anywhere in the reference.

d. Make sure the scales are the same on both x and y axes. I believe this may already be the case, which is great. This is important for interpreting the "steepness" of the diagonals.

(a) We appreciate this important point and have clarified in the methods and figure legend that it is an alignment of the 200bp length-filtered assemblies.

(b) The X-axis consistently scales across the plots with Y-axis in 1:1 relation, but as noted previously missing sequences do not show up on the Oxford Grid (however we do list the missing percentages on the panels). The apparent change in x-axis is an optical illusion caused by either duplications or deletions. All alignments are on a 45 degree angle, but some are so short, and are overlapped by other (short) alignments, for example, that the slope appears steeper than 45 degrees when you look at the figure (as described by the reviewer). Also as described by the reviewer, deletions cause an apparent gentler slope than 45 degrees as individual alignments spread across the reference with gaps between. But within each alignment itself, scales are consistent for X and Y axes and the diagonal line is at precisely 45 degrees. The apparent deviation occurs because 1) many of the alignments are very short, 2) there are lots of duplications (leading to steeper apparent overall slope) and 3) there are lots of deletions (leading to shallower apparent overall slope).

(c) Unfortunately there is a well-known challenge of showing full genomes in LAST and that is to make sure that everything can be seen. The reviewer requests that the non-matching portions be shown. This is not practical because it would produce huge stretches of blank space at the end of the oxford grid that make it harder to see the patterns within the matching regions. Many users of LAST have discussed this issue but no standard method of including non-matching sequences has been adopted by the field because it adds very little to the visualization and detracts from the interpretation of the results. We therefore have left the plots as shown but now include the quantitative % missing and % duplicated sequence (also shown in Table S2) to make explicit what isn't shown. These percentages relate directly to the apparent slope of the line as discussed in our response to comment (b).

(d) The reviewer is correct that the scale is the same on both x and y, and as we discussed above.

Have all data underlying the figures and results presented in the manuscript been provided?

Large-scale datasets should be made available via a public repository as described in the *PLOS Computational Biology* data availability policy, and numerical data that underlies graphs or summary statistics should be provided in spreadsheet form as supporting information.

Reviewer #1: Yes

Reviewer #2: Yes

PLOS authors have the option to publish the peer review history of their article (what does this mean?). If published, this will include your full peer review and any attached files.

If you choose "no", your identity will remain anonymous but your review may still be made public.

Do you want your identity to be public for this peer review? For information about this choice, including consent withdrawal, please see our Privacy Policy.

Reviewer #1: No

Reviewer #2: No

Figure Files:

While revising your submission, please upload your figure files to the Preflight Analysis and Conversion Engine (PACE) digital diagnostic tool, https://pacev2.apexcovantage.com. PACE helps ensure that figures meet PLOS requirements. To use PACE, you must first register as a user. Then, login and navigate to the UPLOAD tab, where you will find detailed instructions on how to use the tool. If you encounter any issues or have any questions when using PACE, please email us at figures@plos.org.

Data Requirements:

Please note that, as a condition of publication, PLOS' data policy requires that you make available all data used to draw the conclusions outlined in your manuscript. Data must be deposited in an appropriate repository, included within the body of the manuscript, or uploaded as supporting information. This includes all numerical values that were used to generate graphs, histograms etc.. For an example in PLOS Biology see here: http://www.plosbiology.org/article/info%3Adoi%2F10.1371%2Fjournal.pbio.1001908#s5.

Reproducibility:

To enhance the reproducibility of your results, PLOS recommends that you deposit laboratory protocols in protocols.io, where a protocol can be assigned its own identifier (DOI) such that it can be cited independently in the future. For instructions see http://journals.plos.org/ploscompbiol/s/submission-guidelines#loc-materials-and-methods
